# Diagnostically Challenging Case: Metastatic Hepatocellular Carcinoma With No Liver Lesion at Imaging

**DOI:** 10.1200/JGO.17.00009

**Published:** 2017-05-24

**Authors:** Angela R. Zambrano, Juan C. Quesada, Ana M. Torres, Juliana Escobar, Manju L. Prasad, Martín E. Renjifo, Luz M. Pabón

**Affiliations:** **Angela R. Zambrano**, **Juan C. Quesada**, **Ana M. Torres**, **Juliana Escobar**, **Martín E. Renjifo**, and **Luz M. Pabón**, Fundación Valle del Lili, Cali, Colombia; and **Manju L. Prasad**, Yale University School of Medicine, New Haven, CT.

## CASE REPORT

A 72-year-old man with a history of chronic alcoholism and cirrhosis Child score A was referred to the oncology department of a tertiary hospital in Cali, Colombia, for assessment of a growing mass in his oral cavity. Additionally, a mass located on the left adrenal gland was detected during the surveillance cirrhosis controls. On examination, an exophytic lesion of approximately 10 cm on the left mandible was noted. He was hospitalized so laboratory tests and procedures could be performed to establish the primary cancer diagnosis and treatment.

**Fig 1:**
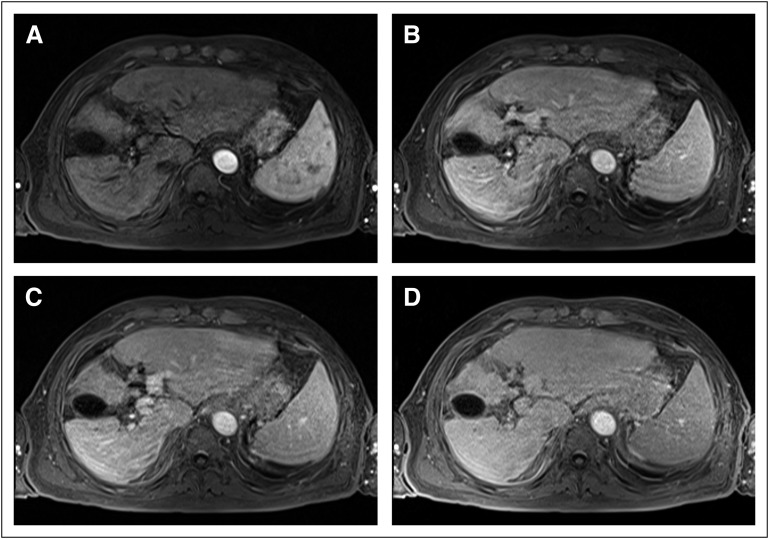
Axial sequence volumetric interpolated breath-hold examination T1-weighted images. (A) Early arterial, (B) arterial, (C) portal venous, and (D) equilibrium phases. Altered hepatic segmentation and contours compatible with chronic liver disease; diffuse changes are shown in the enhancement pattern of focal lesions with no determination of hypervascular behavior.

**Fig 2:**
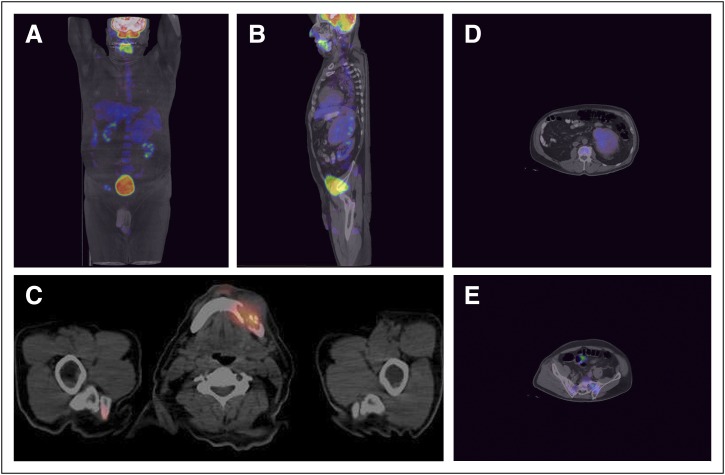
[^18^F]Fluorodeoxyglucose positron emission tomography (PET)–computed tomography (CT) scan. (A) coronal and (B) saggital whole-body images showed (C) expansile lytic hypermetabolic lesion on the left side of mandible associated with (D) left adrenal hypermetabolic mass and (E) lytic hypermetabolic lesions on the right iliac bone and sacrum. Heterogeneous hepatic distribution of radiotracer without visualization of focal lesions on PET and CT images.

Serologic tests were negative for hepatitis B and C panels. Serum alpha-fetoprotein (AFP) and carcinoembryonic antigen were not significantly altered. Abdomen magnetic resonance imaging (MRI) and computed tomography (CT) scan showed changes consistent with cirrhosis; no hypervascular changes or signs of hepatocellular carcinoma (HCC) were detected (Fig 1). Positron emission tomography–CT detected abnormal hypermetabolic activity in the mandible corresponding to the mass, pelvis bones, and adrenal mass, suggesting neoplastic lesions, but no significant activity in the liver (Fig 2). Biopsies of adrenal and mandible lesions showed morphology and immunohistochemistry consistent with hepatoid differentiation in carcinoma. These findings did not, however, correlate with the imaging evidence.

The history of cirrhosis along with the hepatoid characteristics of the adrenal and mandibular tumors suggested metastatic HCC. However, the absence of a liver tumor on imaging raised the possibility of an adrenocortical carcinoma with hepatoid differentiation, a rare tumor with an even rarer presentation.^1,2^

The patient experienced progression 1 year later despite two treatment lines. At this time, a decision to perform an exploratory laparoscopy with liver biopsy was made. At laparoscopy, the liver was cirrhotic and diffusely nodular without a dominant mass. Numerous representative biopsies were taken from different areas. They revealed histologic and immunophenotypic findings of infiltrative HCC.

Tumor markers were taken serially; initial AFP was 9.4 ng/mL (normal values, < 10.0 ng/mL), and during follow-up, AFP values were as follows: 7.3, 3.17, and 4.8 ng/mL. Carcinoembryonic antigen values were < 0.5 ng/mL (normal values, < 3.0 ng/mL). Both tumor markers were considered nonsignificant.

**Fig 3:**
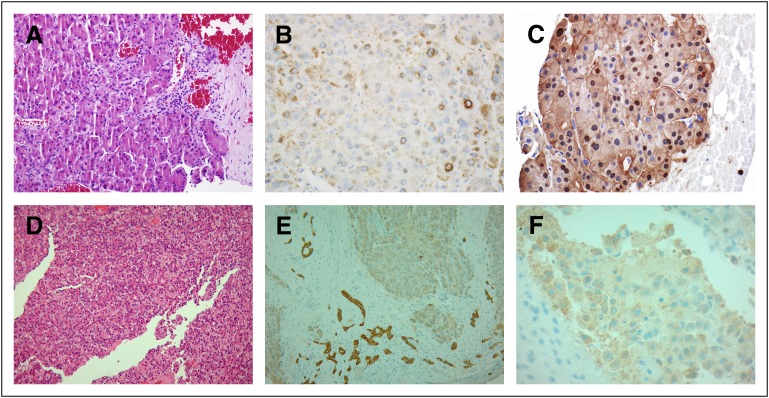
(A) Mandible biopsy (hematoxylin and eosin [HE] ×20). The biopsy was composed entirely of large polygonal oncocytic cells arranged in trabeculae and clusters. (B) Left adrenal core biopsy (Hep-Par-1 immunohistochemical stain). (C) Left adrenal core biopsy (arginase-1 [Arg-1] immunohistochemical stain). The tumor was positive for Hep-Par-1 and Arg-1, consistent with adrenal gland with hepatoid differentiation. (D) Liver biopsy (HE). Histologic sections show loss of tissue architecture, with hepatocytes arranged in trabeculae or acini. (E) Liver biopsy (cytokeratin AE1/AE3). Diffusely and weakly positive immunostain. (F) Liver biopsy. (Hep-Par-1 immunohistochemical stain). Granular cytoplasmic staining pattern.

The adrenal biopsy reported that no normal adrenal gland tissue was seen. Regarding the immunohistochemical markers, the tumor was focally positive for pancytokeratin, Hep-Par-1, and arginase-1 (Arg-1), consistent with hepatoid differentiation. The tumor cells were negative for AFP, glypican-3, and thyroglobulin. All adrenal markers, including inhibit, melon-A, calretinin, and podoplanin, were negative. The proliferation index was high (Ki-67), and CD10 highlighted a canalicular pattern within the tumor (Fig 3). The mandibular biopsy showed morphology and immunoprofile results similar to those of the adrenal tumor (Fig 3). The report concluded with a diagnosis of oncocytic carcinoma with hepatoid features, and clinical and radiologic correlation was recommended.

RNA in situ hybridization for albumin was performed using RNAview (Affymetrix, Cambridge, MA) on the adrenal core biopsy and was positive, confirming hepatoid differentiation and suggesting that the tumor was either from the liver or an unusual hepatoid variant of adrenal cortical carcinoma.

The liver biopsy showed a neoplastic lesion composed of hepatocytic cells arranged as tubules and rosettes. Immunohistochemistry analysis showed positive results for cytokeratin AE1/AE3 and HePar-1. The markers C7-C20 and AFP were negative. The proliferation index (Ki-67) was 30%. The report concluded that the histology and immunochemistry markers were consistent with a diagnosis of HCC (Fig 3).

## DISCUSSION

Here we discuss a case of HCC presenting as metastatic tumor in the mandible and adrenal gland, without evidence of a dominant hepatic primary lesion on imaging. The patient had as first clinical manifestation a mandibular mass and was subsequently found to have a left adrenal tumor, both with hepatoid differentiation. Multiple imaging tests (CT scan, MRI, and positron emission tomography–CT) failed to detect a definitive liver tumor, and serum biomarkers for HCC remained negative. From this point on, some hypotheses were proposed, the first one being metastatic HCC. Although < 1% of cases of HCC involve oral metastases^3^ and approximately 5% of HCCs may initially present as extrahepatic metastases,^4^ it is important to note that, in the group of patients with HCC metastasis to the oral cavity, in approximately 66% of cases, a metastatic oral tumor is noted before the primary hepatic lesion.^5^

The second hypothesis regarding a possible diagnosis was a hepatoid carcinoma of the adrenal gland. This type of cancer is extremely rare, with histopathologic features that mimic those of HCC. It is aggressive and tends to raise serum markers such as AFP^1,3^; however, serum markers and immunohistochemistry analysis were negative in our patient.

The current guidelines^6,7^ state that the diagnosis of HCC in cirrhotic patients should be confirmed based on imaging or, less frequently, on biopsy analyses. Imaging criteria consist of detection by at least three-phase contrast-enhanced CT or MRI of the HCC radiologic hallmark,^6^ characterized by intense arterial uptake or enhancement followed by contrast washout or hypointensity in the delayed venous phase.^6^ Liver lesions < 1 cm should be evaluated by at least three-phase contrast-enhanced CT or MRI every 3 to 6 months. Liver lesions > 1 cm should first be evaluated by one of the two imaging techniques mentioned. A finding of two classic enhancements is considered to be diagnostic of HCC.^6^ The radiologic pattern identifies HCCs with limited levels of sensitivity but up to 100% specificity, depending on the size of the liver nodules detected.^8^ Biopsy analyses are recommended for focal hepatic lesions with atypical imaging features or ambiguous findings on CT or MRI, for lesions > 1 cm if only one or nonclassic enhancement patterns are present on the results of the two imaging techniques, or for lesions detected in the absence of cirrhosis.^6,9^ The biggest difficulty in our patient case was the lack of findings suggestive of HCC on imaging, which is the main diagnostic criterion. According to a recent meta-analysis and systematic review,^10^ in which the test performance of imaging techniques for the detection of HCC was measured, in nonsurveillance settings, CT had a sensitivity of 83% and a specificity of 91% for HCC diagnosis. MRI had a sensitivity and specificity of 86% and 89%, respectively.

Liver biopsy can enable diagnosis of HCC in 70% to 90% of cases,^11^and immunohistochemistry analysis should be used as an ancillary tool in the diagnosis of HCC. The triad of Arg-1, Hep-1, and glypican-3 has been recommended as the most effective method of determining metastatic carcinoma HCC, with Arg-1 being the most specific marker.^12^ Mandible and adrenal gland samples were positive for Hep-Par-1 and Arg-1, supporting the hepatoid nature of these lesions.

Genetic analysis consisted of detecting albumin in the adrenal gland biopsy via RNA in situ hybridization, which confirmed the hepatoid characterization of the sample. This test is considered highly sensitive in poorly differentiated HCC versus immunohistochemistry, and the combination of Arg-1 and genetic analysis significantly improves diagnostic accuracy.^13^

Finally, several liver biopsies were taken, and their pathologic analysis revealed a morphologic and immunohistochemical pattern compatible with infiltrative metastatic HCC. This subtype is characterized by a diffuse and ill-defined phenotype that corresponds to 7% to 20% of cases of HCC. It is considered a diagnostic challenge because of the difficulty of distinguishing, in the imaging, cancerous cells from background changes in cirrhosis. Whereas for nodular HCC the radiologic criteria are defined, for infiltrative HCC they are unclear, because imaging techniques fail to detect this subtype in approximately 40% of cases.^4,14,15^ The macroscopic pattern is extrapolated to images, with a permeative and ill-defined appearance, which present an inconsistent uptake in the arterial phase, reported as minimal or as patches that can be visualized as iso- or hypointense. A diagnostic key for this subtype of HCC is the presence of portal vein thrombosis, which is found to be present in 68% of cases.^4,14,15^ However, none of these imaging signs were present on our patient’s images.

To conclude, this is a rare case of HCC presenting with oral and adrenal masses but with no definitive liver tumor on imaging and serologic testing. This case highlights the importance of early pathologic diagnosis of HCC in cases where there is a clinical suggestion of HHC and images are inconclusive. The clinical team must take into account the different morphologic subtypes of HCC, not only the nodular subtype that is widely described by current literature. The infiltrative variant of HCC is diagnostically challenging and may lead to a late diagnosis.
